# Endothelium-Independent Vasodilatory Effect of Sailuotong (SLT) on Rat Isolated Tail Artery

**DOI:** 10.1155/2020/8125805

**Published:** 2020-09-22

**Authors:** S. Y. Yeon, S. W. Seto, G. H. H. Chan, M. Low, H. Kiat, N. Wang, J. Liu, D. Chang

**Affiliations:** ^1^NICM Health Research Institute, Western Sydney University, Penrith, NSW 2751, Australia; ^2^Department of Applied Biology and Chemical Technology, The Hong Kong Polytechnic University, Hung Hom, Kowloon, Hong Kong SAR, China; ^3^Hong Kong Community College, The Hong Kong Polytechnic University, Hung Hom, Kowloon, Hong Kong; ^4^Faculty of Medicine, University of New South Wales, Kensington, NSW 2052, Australia; ^5^School of Medicine, Western Sydney University, Penrith, NSW 2571, Australia; ^6^Faculty of Medicine and Health Sciences, Macquarie University, Sydney, NSW 2113, Australia; ^7^Key Laboratory of Chinese Medicinal Formula of Anhui Province, Anhui University of Chinese Medicine, Hefei 230012, China; ^8^Xiyuan Hospital, China Academy of Chinese Medical Sciences, Beijing, China

## Abstract

**Background:**

Sailuotong (SLT) is a standardized three-herb formulation consisting of extracts of *Panax ginseng*, *Ginkgo biloba*, and *Crocus sativus* for the treatment of vascular dementia (VaD). Although SLT has been shown to increase cerebral blood flow, the direct effects of SLT on vascular reactivity have not been explored. This study aims to examine the vasodilatory effects of SLT and the underlying mechanisms in rat isolated tail artery.

**Methods:**

Male (250–300 g) Wistar Kyoto (WKY) rat tail artery was isolated for isometric tension measurement. The effects of SLT on the influx of calcium through the cell membrane calcium channels were determined in Ca^2+^-free solution experiments.

**Results:**

SLT (0.1–5,000 *μ*g/ml) caused a concentration-dependent relaxation in rat isolated tail artery precontracted by phenylephrine. In the contraction experiments, SLT (500, 1,000, and 5,000 *μ*g/mL) significantly inhibited phenylephrine (0.001 to 10 *μ*M)- and KCl (10–80 mM)-induced contraction, in a concentration-dependent manner. In Ca^2+^-free solution, SLT (500, 1,000, and 5,000 *μ*g/mL) markedly suppressed Ca^2+^-induced (0.001–3 mM) vasoconstriction in a concentration-dependent manner in both phenylephrine (10 *μ*M) or KCl (80 mM) stimulated tail arteries. L-type calcium channel blocker nifedipine (10 *μ*M) inhibited PE-induced contraction. Furthermore, SLT significantly reduced phenylephrine-induced transient vasoconstriction in the rat isolated tail artery.

**Conclusion:**

SLT induces relaxation of rat isolated tail artery through endothelium-independent mechanisms. The SLT-induced vasodilatation appeared to be jointly meditated by blockages of extracellular Ca^2+^ influx *via* receptor-gated and voltage-gated Ca^2+^ channels and inhibition of the release of Ca^2+^ from the sarcoplasmic reticulum.

## 1. Background

Cerebrovascular diseases (CVDs), including ischemic stroke and vascular dementia (VaD), are among the major causes of morbidity and mortality in developing and developed countries [1]. CVDs are strongly associated with a number of risk factors, such as hypertension, obesity, aging, diabetes, and hypercholesterolemia [2]. Altered vascular reactivity, such as impaired vasodilatation or enhanced vasoconstriction, has been considered one of the common characteristics associated with these risk factors [3–5], directly contributing to the development and progression of CVD. For example, impaired vascular relaxation has been shown to play a role in the development of VaD in deoxycorticosterone acetate (DOCA) salt-induced hypertensive rats [6]. Similarly, impaired carotid artery relaxation has been suggested as a contributor to stroke and dementia in aged mice [7]. Therefore, interventions that can induce vasodilation or suppress vasoconstriction could be useful in reducing the progression of CVD.

Sailuotong (SLT) is a standardized three-herb formula Chinese herbal medicine designed for the management of VaD associated with ischemic stroke [8, 9]. The SLT formula consists of standard extracts of specific dosages of *Panax ginseng* C. A. Meyer (ginseng), *Gingko biloba* L. (ginkgo), and *Crocus sativus* L. (saffron). The chemical profile and ratio of the three herbal extracts had been determined and studied in detail previously [10, 11]. The cognitive enhancing effect of SLT has been demonstrated in both preclinical and clinical studies. In a chronic cerebral hypoperfusion model induced by bilateral common carotid artery ligation in rats, eight-week treatment of SLT significantly suppressed hypoperfusion-induced cognitive impairments, and this change is associated with reduction of activity of cholinesterase and increased acetylcholine (ACh) levels and superoxide dismutase (SOD) activity the brain tissue [8]. Our previous work also showed that SLT protected cultured human vascular endothelial cells against hydrogen peroxide-induce injury via increase in SOD activity [12]. The potential beneficial effects of SLT for VaD were demonstrated in two separate phase II randomized, double-blinded, placebo-controlled clinical studies. In these studies, treatment with SLT was shown to significantly improve cognitive function and cerebral blood flow to the inferior frontal and anterior temporal lobes in patients with VaD [11, 13].

Reports from animal and clinical studies have indicated that the clinically beneficial effect of SLT is, at least, partially associated with an increase in cerebral blood flow [14, 15]. However, the underlying mechanism(s) associated with these SLT-mediated cerebral blood flow increase remain to be determined. In this study, we hypothesised that SLT has direct modulatory effects in vascular reactivity. Therefore, this study was aimed to evaluate the vascular effects and the underlying mechanisms of SLT using rat isolated tail artery.

## 2. Methods

### 2.1. Chemical and Drugs

SLT extracts were provided in-kind by the Shineway Pharmaceutical Group (Shijiazhuang, China). Acetylcholine (ACh), phenylephrine, calcium chloride (CaCl_2_), tetraethylammonium (TEA), clotrimazole, glibenclamide, potassium chloride (KCl), ethylene glycol-bis(*β*-aminoethyl ether)-*N*,*N*,*N*′,*N*′-tetraacetic acid (EGTA), *l*-*N*-nitro arginine methyl ester (L-NAME), and nifedipine were purchased from Sigma-Aldrich (St Louis, MO, USA). All the other reagents were of analytical grade.

### 2.2. High-Performance Thin-Layer Chromatography (HPLC) Analysis of the SLT Formula

HPLC-PDA was employed to profile the phytochemical composition of the SLT extract used in the study. The HPLC-PDA analysis was performed on a Prominence-I LC-2030 3D Plus Shimadzu HPLC system controlled by Lab Solutions software (Shimadzu, Australia). Separation was achieved using a Shimadzu Shim-pack GIST (Shimadzu, Australia) reverse phase C18 column (4.6 × 150 mm I.D., 5 *μ*m) maintained at 40°C.

The SLT extract was dissolved by sonication in 30% aqueous acetonitrile for 30 min at 5 mg/ml. Individual solutions of standards, crocin, ginsenoside Re, ginsenoside Rg1, ginsenoside Rd, quercetin, kaempferol, and isorhamnetin were prepared to 1 mg/10 ml in 30% aqueous acetonitrile for identification and combined for analysis. The sample and mixed standard were syringe-filtered with 0.45 *μ*m PTFE.

The SLT HPLC-PDA profiles were generated by 20 *μ*l injection. The mobile phase consisted of 0.1% (v/v) aqueous formic acid (mobile phase A) and 0.1% (v/v) formic acid in acetonitrile (mobile phase B). The gradient program was 10% B for 1 min with a linear increase, to 45% B at 45 min, and then, a wash and re-equilibration. The mobile phase flow rate was maintained at 1.1 ml/min. The PDA was set to acquire absorbance data from 190 to 800 nm.

### 2.3. Animals

A total of forty 12-week-old, male Wistar Kyoto (WKY) rats weighting 200–250 g were obtained from the Animal Resource Centre (Canning Vale, Western Australia). All experimental animals were housed under a 12:12 hour light-dark cycle (relative humidity: 50–60%; temperature: 22 ± 1°C) and were given standard chow and water *ad libitum* throughout the experimental period. On the day of experiment, the rats were sacrificed by CO_2_ asphyxiation, and vascular preparations were isolated under a dissection microscope. All animal protocols conformed to the Guide for the Care and Use of Laboratory Animals by the Australian Code of Practice for the Care and Use of Animals for Scientific Purpose [16]. Institutional ethics approval (Approval number: A12041) was obtained from Western Sydney University prior to commencement of the study.

### 2.4. Isometric Tension Measurement

Isometric force measurements and experimental protocols were performed on tail artery isolated from the WKY rats as described previously with slight modifications [5, 17]. The isometric force of the arterial ring segments was measured using a multiwire myograph system (Model 620M; Danish Myo Technology, DMT, Denmark). During the isometric tension measurement, the chambers of the myograph were filled with Krebs–Henseleit solution (mmol/L: NaCl 118.0, KCl 4.7, K_3_PO_4_ 1.2, MgSO_4_ 1.2, NaHCO_3_ 25.0, CaCl_2_ 1.8, glucose 11.0), unless stated otherwise. The Krebs–Henseleit solution were prepared and used as follows: All solutions were warmed to 37°C and bubbled with carbogen (95% O_2_ and 5% CO_2_) before they were added to the myograph chamber. Each chamber containing 5 ml of Krebs buffered solution was bubbled with 95% O_2_ and 5% CO_2_ and maintained at a pH of 7.4 at 37°C throughout the experiment. Arterial rings (1.5–2.0 mm length) were mounted in the organ chambers, and normalized passive resting force and the corresponding diameter were determined for each preparation from its own length-pressure curve and stretched to their optimal lumen diameter (i.e.,∼90% of the diameter which is equivalent to a transmural pressure of 100 mm Hg). The rings were, then, left for a stabilization period of about 30 minutes until a stable base line tone was obtained before beginning of the experiment. The presence of the endothelium was tested with acetylcholine (1 *μ*M) after preconstriction with phenylephrine (PE) (1 *μ*M). Tissues which showed a relaxation to acetylcholine of less than 60% of the phenylephrine contraction were not considered with intact endothelium and, therefore, not employed in this study. Responses were recorded using computerized data acquisition and recording software.

### 2.5. Experimental Protocols of Vascular Tension Studies

#### 2.5.1. Protocol 1: Vasodilatory Effect of the SLT Extract in Rat Isolated Tail Arteries

The acute and direct vasodilatory effect of SLT was evaluated on rat isolated tail artery. PE (1 *μ*M) was applied to induce a steady contraction. When the contraction reached a plateau, the SLT extract (0.1–5000 *μ*g/ml) was added cumulatively to the isolated tail artery rings with or without the presence of L-NAME (20 *μ*M) to obtain a concentration-response curve. The relaxation effect was calculated as the percentage of the contraction in response to PE.

#### 2.5.2. Protocol 2: Effects of Potassium Channel Blockers on SLT-Induced Vasodilation in Rat Isolated Tail Arteries

Rat isolated tail arteries were preincubated with various K^+^ channel blockers: (1) nonselective K^+^ channel blocker, tetraethylammonium (TEA, 1 mM); (2) selective ATP-sensitive K^+^ channel (K_ATP_) blocker, glibenclamide (3 *μ*M), and (3) Ca^2+^-dependent K^+^ channel (K_Ca_) blockers, clotrimazole (5 *μ*M). Each blocker was allowed to incubate with the preparation for 30 min before the construction of a concentration-response curve.

#### 2.5.3. Protocol 3: Calcium-Induced Vasoconstriction in Rat Isolated Tail Arteries

The effect of SLT on calcium influx induced by two different agents was evaluated. The preparations were initially contracted with either KCl (80 mM) or PE (10 *μ*M) to determine maximum contraction of each preparation in normal Krebs solution. The preparations were washed with a calcium-free solution (identical concentration of EGTA substituted for Ca^2+^ in a normal Krebs solution) until fully relaxed. To exhaust the intracellular calcium storage, the preparations were stimulated with either PE (10 *μ*M) or KCl (80 mM) repeatedly, with calcium-free solution wash in between, until any contractile response disappeared. The preparations were, then, incubated with SLT at various concentrations (500, 1,000, and 5,000 *μ*g/ml) for 30 min. After incubation, a single dose of the contractile agent (either KCl (80 mM) or PE (10 *μ*M) was added to the chamber, and calcium (0.001–3 mM)-induced contraction concentration response curves were constructed.

#### 2.5.4. Protocol 4: Phenylephrine- and Potassium Chloride- (KCl-) Induced Vasoconstriction in Rat Isolated Tail Arteries

In order to assess the effects of SLT on the contraction induced by phenylephrine and potassium chloride (KCl), concentration-response curves of PE and KCl were constructed with or without the presence of SLT at various dosages (500, 1,000, and 5,000 *μ*g/ml). The arterial preparations were allowed to preincubate with SLT (0, 500, 1,000, and 5,000 *μ*g/ml) for 30 minutes' prior the construction of the PE or KCl-induced vasoconstriction concentration-response curve.

#### 2.5.5. Protocol 5: Role of Intracellular Calcium-Induced Vasoconstriction in Rat Isolated Tail Arteries

To clarify the role of intracellular Ca^2+^ release from the sarcoplasmic reticulum (SR) in the relaxant effect of SLT, the isolated tail artery rings were exposed to a Ca^2+^-free solution for 15 minutes before the application of 1 *μ*M of PE to induce the first transient contraction (Con 1). The rings were, then, washed with a normal Krebs solution for three times and incubated in the normal Krebs solution for 45 minutes to allow refill of the intracellular Ca^2+^ storage. After the 45-minute incubation, the medium was rapidly replaced with a Ca^2+^-free solution and allowed to incubate for 15 minutes. The second contraction (Con 2) was induced by 1 *μ*M of PE with or with the presence of SLT (500, 1,000, or 5,000 *μ*g/ml). The ratio of Con 2 to Con 1 was calculated.

### 2.6. Statistical Analysis

Data are expressed as means ± S.E.M., and *n* denotes the number of replications for each data point. Relaxation was expressed as the percentage of the contraction elicited by phenylephrine or KCl. Statistical comparisons were performed using the *t*-test or two-way analysis of variance (ANOVA), where appropriate. Differences were considered to be statistically significant at *P* < 0.05. All statistical analyses were performed using GraphPad Prism 5 software (GraphPad Software, Inc., USA).

## 3. Results

### 3.1. High-Performance Thin-Layer Chromatography (HPLC) Analysis of the SLT Extract

**Figure 1:**
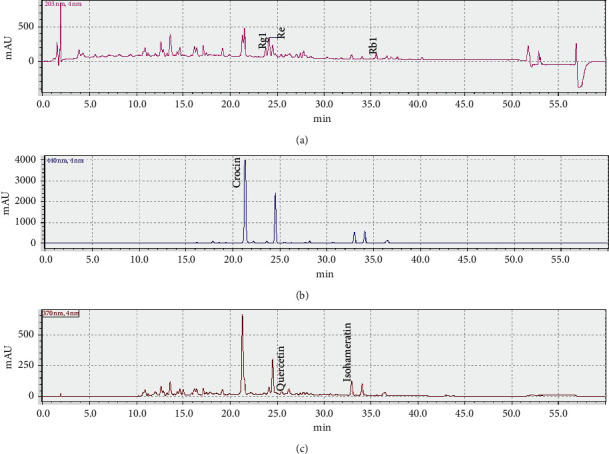
HPLC chromatograms of the SLT extract. The chromatogram (a) at 203 nm (pink) shows the ginsenosides Rg1, ginsenosides Re, and ginsenosides. The chromatogram (b) at 440 nm (blue) shows crocin. The chromatogram (c) at 370 nm (maroon) shows quercetin and isorhamnetin.

The active ingredients and contents in SLT have been reported in details previously [10, 11]. Six major bioactive compounds were analyzed using HPLC. Similar to these studies, our results showed that all these major bioactive compounds, including ginsenosides Rg1, ginsenosides Re, ginsenosides Rb1, quercetin, isorhamnetin, and crocin, are present in the SLT extract (Figure 1). The chromatogram is visualized at different wavelengths corresponding to the optimal wavelength to detect the relevant group of markers. SLT is reported to contain ginsengs as ginsenosides Rg1 (2.4 mg/capsule), ginsenosides Re (1.6 mg/capsule), and ginsenosides Rb1 (4.49 mg/capsule). The ginsenosides were confirmed to be present in similar concentrations by matching retention time and the UV spectrum to the reference standards as shown in Figure 1 at 203 nm. The peak size is not reflective of abundance as the different ginsenosides have unique molar absorptivity. SLT is reported to contain *Ginkgo biloba*, with markers quercetin (2.71 mg/capsule) and isorhamnetin (1.55 mg/capsule). These were identified in the SLT extract used in this study as shown in Figure 1 at 370 nm. SLT is reported to contain saffron, with the marker crocin (1.7 mg/capsule). Crocin was identified in the SLT extract used in this study as shown in Figure 1 at 440 nm. The HPLC analysis of the SLT extract supplied by Shineway was consistent with other reports [10, 11].

### 3.2. Vasodilatory Effects of the SLT Extract in Rat Isolated Tail Arteries

**Figure 2:**
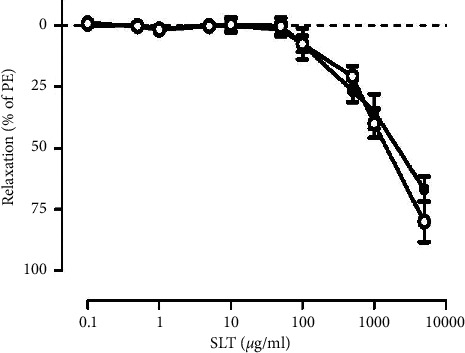
Cumulative concentration-response of the SLT extract (0.1–5,000 *μ*g/ml) on phenylephrine (1 *μ*M)-preconstricted rat isolated tail artery with (●) or without (○) the presence of L-NAME (20 *μ*M). Values are expressed as mean ± SEM (*n* = 6).

SLT (0.1–5,000 *μ*g/ml) caused a relaxation in the PE (1 *μ*M)-preconstricted rat isolated tail artery in a concentration-dependent manner. The relaxation was initiated at 100 *μ*g/ml and achieved 80.104 ± 9.80% at 5,000 *μ*g/ml (*n* = 6) (Figure 2). Preincubation of the isolated tail artery with L-NAME (20 *μ*M) did not significantly alter the SLT-induced relaxation (*n* = 6) (Figure 2).

### 3.3. Effects of Potassium Channel Blockers on SLT-Induced Vasodilatation in Rat Isolated Tail Arteries

**Figure 3:**
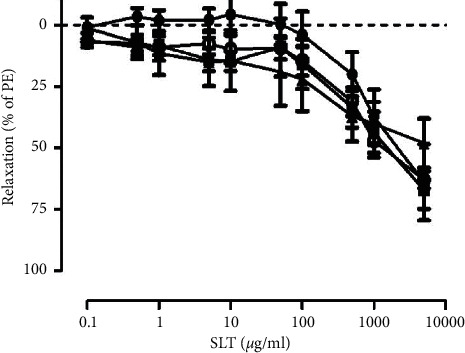
Effects of potassium channel blockers on SLT extract-induced relaxation on phenylephrine (1 *μ*M)-preconstricted rat isolated tail artery. Tail arteries were preincubated with either vehicle control (○), tetrethyl-ammonium (TEA, a nonselective potassium channel blocker) (1 mM) (●), glibenclaminde (Glib, an ATP-sensitive potassium channel blocker) (3 *μ*M) (■), or clotrimazole (KCa, a calcium-activated potassium channel blocker) (5 *μ*M) (▲) for 30 minutes before construction of the concentration-response curve. Values are expressed as mean ± SEM (*n* = 5-6).

Opening of potassium channels plays an important role in vasodilatation. We examined the involvement of potassium channels on the SLT-induced relaxation in the rat isolated tail arteries. Tetraethylammonium (TEA, a nonselective potassium channel blocker) (1 mM), glibenclamide (Glib, an ATP-sensitive potassium channel blocker) (3 *μ*M), and clotrimazole (K_Ca_, a calcium-activated potassium channel blocker) (5 *μ*M) were tested. As shown in Figure 2, preincubation of potassium channel blockers did not significantly alter the SLT-induced vasodilatation in PE (1 *μ*M)-preconstricted rat isolated tail artery (*n* = 5-6) (Figure 3).

### 3.4. Effects of the SLT Extract on Calcium-Induced Vasoconstriction in Rat Isolated Tail Arteries Stimulated by Phenylephrine or KCl

**Figure 4:**
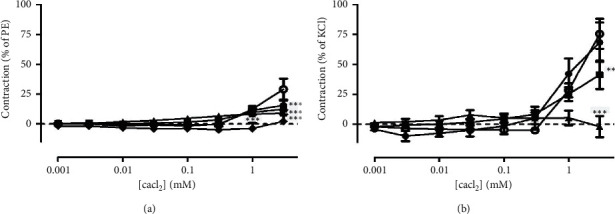
(a) Effect of the vehicle control (○), SLT extract (500 (●), 1000 (■), or 5000 (▲) *μ*g/ml), or nifedipine (10 *μ*M) (♦) on Ca^2+^-induced vasoconstriction in phenylephrine (PE) (10 *μ*M)-stimulated rat isolated tail arteries. Values are expressed as mean ± SEM (^*∗∗∗*^*P* < 0.001 vs Control) (*n* = 5). (b) Effect of the vehicle control (○) and SLT extract (500 (●), 1,000 (■), or 5,000 (▲) *μ*g/ml) on Ca^2+^-induced vasoconstriction in potassium chloride (KCl) (80 mM)-stimulated rat isolated tail arteries. Values are expressed as mean ± SEM (^*∗∗*^*P* < 0.01 vs. control ^*∗∗∗*^*P* < 0.001 vs. control) (*n* = 5–8).

The effect of the SLT extract on calcium-influx-induced vasoconstriction on rat isolated tail arteries was assessed with two vasocontractile stimulants (phenylephrine and KCl) by reintroducing calcium (0.001–3 mM) into the calcium-free buffer. In the phenylephrine (10 *μ*M)-stimulated tail arteries, cumulative administration of calcium chloride (0.001–3 mM) induced a vasoconstriction in a concentration-dependent manner, with 29.167 ± 7.23% contraction observed at 3 mM (*n* = 5). Preincubation of the SLT extract (500, 1,000, and 5,000 *μ*g/ml) dose-dependently suppressed the calcium-induced vasoconstriction in the phenylephrine (10 *μ*M)-isolated tail arteries. SLT at 5,000 *μ*g/ml and nifedipine (10 *μ*M) markedly suppressed the calcium-induced vasoconstriction (Figure 4(a)).

Similarly, in the KCl (80 mM)-stimulated tail arteries, cumulative administration of calcium chloride (0.001–3 mM) induced a vasoconstriction in the tail artery in a concentration-dependent manner, with 75.460 ± 14.52% contraction observed at 3 mM (*n* = 6). Preincubation of the SLT extract at 1,000 and 5,000 *μ*g/ml, but not 500 *μ*g/ml, markedly inhibited the calcium-induced vasoconstriction in the KCl (80 mM)-stimulated isolated tail arteries. SLT at 5,000 *μ*g/ml abolished the calcium-induced vasoconstriction (*n* = 6) (*P* < 0.001) (Figure 4(b)).

### 3.5. Effects of the SLT Extract on Phenylephrine- and KCl-Induced Vasoconstriction in Rat Isolated Tail Arteries

**Figure 5:**
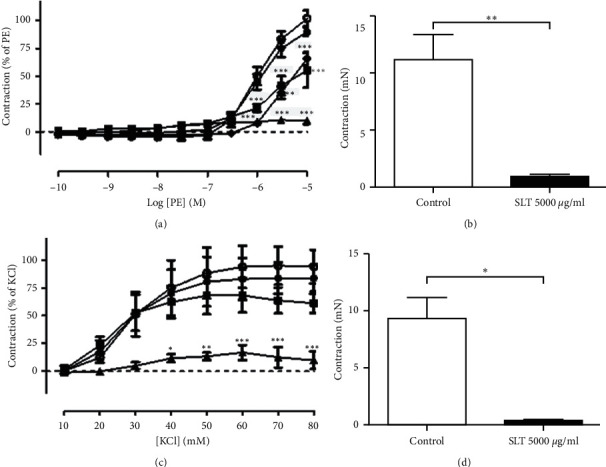
(a) Effect of the vehicle control (○), SLT extract (500 (●), 1000 (■), or 5000 (▲) *μ*g/ml), or nifedipine (10 *μ*M) (♦) on phenylephrine-induced vasoconstriction in rat isolated tail arteries. Values are expressed as mean ± SEM (^*∗∗∗*^*P* < 0.001 vs. control) (*n* = 5). (b) Isometric tension change of phenylephrine (10 *μ*M)-induced vasoconstriction in rat isolated tail arteries with (closed bar) or without (open bar) the presence of the SLT extract (5000 *μ*g/ml). Values are expressed as mean ± SEM (^*∗∗*^*P* < 0.01) (*n* = 8). (c) Effect of the vehicle control (○) or SLT extract (500 (●), 1,000 (■), or 5,000 (▲) *μ*g/ml) on potassium chloride (KCl) (80 mM)-induced vasoconstriction in rat isolated tail arteries. Values are expressed as mean ± SEM (^*∗*^*P* < 0.05; ^*∗∗*^*P* < 0.01; ^*∗∗∗*^*P* < 0.001 vs. control) (*n* = 3). (d) Isometric tension change of KCl (80 mM)-induced vasoconstriction in rat isolated tail arteries with (closed bar) or without (open bar) the presence of the SLT extract (5,000 *μ*g/ml). Values are expressed as mean ± SEM (^*∗∗*^*P* < 0.01) (*n* = 3).

The effects of the SLT extract (500, 1,000, and 5,000 *μ*g/ml) on phenylephrine-induced vasoconstriction in rat isolated tail arteries were evaluated. Cumulative addition of phenylephrine (0.0001–10 *μ*M) caused a vasoconstriction in the tail arteries in a concentration-dependent manner. Preincubation of the SLT extract at 1,000 and 5,000 *μ*g/ml, but not 500 *μ*g/ml, significantly suppressed the phenylephrine-induced vasoconstriction in the isolated tail arteries (*n* = 5) (*P* < 0.001). Nifedipine (10 *μ*M) markedly reduced the PE-induced vasoconstriction (Figure 5(a)). The vasoconstriction caused by phenylephrine at 10 *μ*M was markedly suppressed by 67.58% with the presence of the SLT extract at 5,000 *μ*g/ml (control: 16.44 ± 2.46 mN vs. SLT: 5.33 ± 1.02 mN) (*P* < 0.05) (*n* = 5) (Figure 5(b)).

The effects of the SLT extract (500, 1,000, and 5,000 *μ*g/ml) on KCl-induced vasoconstriction in rat isolated tail arteries were evaluated. Cumulative addition of KCl (10–80 mM) caused a vasoconstriction in the tail arteries in a concentration-dependent manner. Preincubation of the SLT extract at 500, 1,000, and 5,000 *μ*g/ml significantly suppressed the KCl-induced vasoconstriction in the isolated tail arteries in a dose-dependent manner. (*n* = 3) (*P* < 0.001) (Figure 5(c)). The vasoconstriction caused by 80 mM was abolished with the presence of the SLT extract at 5,000 *μ*g/ml (control: 7.90 ± 1.75 mN vs. SLT: 0.54 ± 0.31 mN) (*P* < 0.05) (*n* = 3) (Figure 5(d)).

### 3.6. Effect of SLT on Intracellular Calcium Release

**Figure 6:**
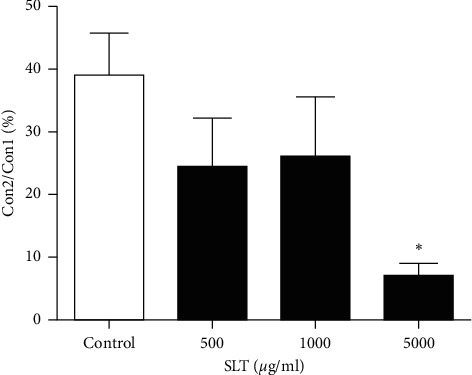
Effect of the SLT extract (500, 1,000, and 5,000 *μ*g/ml) on phenylephrine (10 *μ*M)-induced transient vasoconstriction in rat isolated tail arteries. Con2/Con1 (%) refers to the ratio of the second contraction to the first contraction. Values are expressed as mean ± SEM (^*∗*^*P* < 0.05 vs control) (*n* = 6).

Phenylephrine (PE) (1 *μ*M) caused a transient contraction in the Ca^2+^-free solution through a release of intracellular Ca^2+^ from the sarcoplasmic reticulum. A second transient contraction was induced with or without the presence of SLT (500, 1,000, and 5,000 *μ*g/ml). SLT significantly suppressed the PE-induced transient contraction ratio (Con 2/Con 1) at 5,000 *μ*g/ml (CLT: 39.12 ± 6.28 vs. SLT 5,000 *μ*g/ml: 6.86 ± 1.52) (*P* < 0.05) (*n* = 6) (Figure 6).

## 4. Discussion

Vascular dementia (VaD) is considered to be caused by chronic cerebral ischemia which induces neuronal damage, resulting in a decline in cognitive function eventually. While the development and progression of cognitive impairment and VaD are certainly multifactorial, it has been suggested that cerebrovascular dysfunction leads to reduction of cerebral blood flow which is closely associated with cognitive impairment in diabetes [18, 19]. Sailuotong (SLT) is a standardized three-herb formula designed for the management of VaD. Although data from animal and clinical studies have indicated that the clinically beneficial effect of SLT is closely associated with an increase in cerebral blood flow [11–13], the acute and direct modulatory effects and the underlying mechanisms of actions of SLT in vascular reactivity have not been studied. Results from the current work provide evidence that the SLT extract induces vascular relaxation in rat isolated tail arteries. Furthermore, our results showed that this SLT-induced vasodilatation is mediated *via* endothelium-independent mechanisms including inhibition of extracellular Ca^2+^ influx and release of Ca^2+^ storage from the sarcoplasmic reticulum.

The individual herb extracts of SLT have been shown to induce vasodilatation *via* endothelium-mediated mechanisms [20]. For example, crocetin, a carotenoid derived from saffron, has been shown to exert vasomodulatory effects via an endothelium-dependent mechanism [21]. Similarly, previous studies have demonstrated that ginseng produces its vasodilatory effect through eNOS activation in the endothelium [22, 23]. In contrast, our results revealed that inhibition of the endothelium by L-NAME failed to suppress the SLT-induced vasodilatation, indicating that the SLT-induced relaxation is largely mediated by an endothelium-independent mechanism. This relaxation was most likely caused by the complex constituents within the SLT extract which acted directly on the vascular smooth muscle cell (VSMC). For instance, the *Ginkgo biloba* extract (GBE) has been shown to induce vasodilation *via* multiple mechanisms, including inhibition of calcium influx through the L-type calcium channel and the activation of NO release in the rat isolated aorta [24]. Indeed, it has been shown that, while all the *Ginkgo* extract constituents, including bilobalide, ginkgolide A, ginkgolide B, ginkgolide C, quercetin, and rutin, have vasodilating action, each of them can contribute to GBE-induced vasorelaxation with different mechanisms and signaling pathways [25].

Since our results indicate that the SLT-induced relaxation was not mediated *via* the endothelium, other endothelium-independent mechanisms were considered. Vascular tone and reactivity regulation are primarily controlled by the changes of intracellular calcium within the vascular smooth muscle cell (VSMC) [26]. A number of calcium channels and mechanisms are responsible for the increase of the intracellular calcium level in VSMC [27–30], and many herbs and their isolated compounds have been shown to induce vasodilatation *via* inhibition of calcium influx in the VSMC [24]. For instance, the *Ginkgo biloba* extract has been shown to induce aortic relaxation via inhibition of calcium influx through the calcium channel [24]. Similarly, ginsenoside Rg(3), an active ingredient of *Panax ginseng*, has been shown to inhibit the L-type calcium channel in *Xenopus* oocytes in a dose- and voltage-dependent manner [31]. In the current study, reintroduction of calcium to phenylephrine- or KCl-stimulated isolated tail artery bathed in a calcium-free solution caused contractions in a concentration-dependent manner. These results clearly demonstrated the role of calcium influx in vasoconstriction in response to both receptor-mediated (i.e., phenylephrine) and voltage-mediated (KCl) stimulations. SLT dose-dependently inhibited the calcium-influx-induced vessel contraction to both phenylephrine- and KCl-stimulated tail arteries, suggesting SLT was likely to inhibit both receptor- and voltage-mediated calcium influx. In line with this, the ability of SLT to suppress both phenylephrine- and KCl-mediated responses were clearly demonstrated in the phenylephrine- and KCl-induced vasoconstriction experiments, where SLT markedly suppressed the vasoconstriction caused by both agents. Therefore, our results suggested that SLT suppressed calcium influx by inhibition of both the voltage-operated calcium channel (VOCC) and receptor-operated calcium channel (ROCC).

Although the effects of SLT on intracellular calcium storage regulation in VSMC have not been reported previously, the individual components of SLT have been shown to suppress increase of the intracellular calcium level *via* inhibition of both influxes of extracellular Ca^2+^ and release of intracellular Ca^2+^ storage. In our study, SLT inhibited the PE-induced transient contraction in the isolated tail artery, indicating that SLT suppressed the vasoconstriction *via* inhibition of the release of Ca^2+^ from the sarcoplasmic reticulum. Similar to our study, ginsenoside-Rd, a main active constituent of *Panax ginseng*, has been shown to suppress phenylephrine-induced contraction in rat isolated aorta *via* inhibition of both receptor- and store-operated calcium channels in VSMC [22]. Yang et al. [32] have shown that the antiplatelet and antithrombosis properties of saffron were mediated by a mechanism involving inhibition of calcium mobilization via reducing both intracellular calcium release and extracellular calcium influx [32].

Many herbal medicines have been shown to produce their cardiovascular protective effects *via* the opening of potassium channels in VSMCs, such as the ATP-sensitive potassium channel (K_ATP_) and calcium-activated potassium channel (K_Ca_) [33, 34]. For example, Li et al. [35] showed that ginsenosides (GS), an extract of *Panax ginseng,* induced aortic relaxation and is, at least, partly mediated by the opening of the K_Ca_ channel in the VSMC [35]. Similarly, activation of the potassium channel has also been demonstrated in ginsenoside Rg3-mediated endothelium-dependent relaxation in rat isolated aorta [36]. The *Ginkgo* extract has been shown to relax smooth muscle cell via a TEA-sensitive (a nonselective potassium channel blocker) mechanism [37] and opening of the K_Ca_ channel [38], while other studies suggested that *Ginkgo* induces vasodilatation *via* inhibition of calcium influx [24, 39] and activation of eNOS [40]. In this study, preincubation of TEA (a nonselective potassium channel blocker), glibenclamide (an ATP-sensitive potassium channel blocker), and clotrimazole (a calcium-activated potassium channel blocker) did not have any effects on the SLT-induced relaxation in the rat isolated tail artery, suggesting that the relaxation was independent of potassium channels opening. Although the effects of the individual components of SLT on the potassium channel have been reported previously [36], it is not uncommon that Chinese herbal medicine can induce relaxation independent of potassium channel activation. For example, the Danshen aqueous extract and its active component, Salvianolic acid B, have been shown to produce their vasorelaxant effect independent from potassium channel opening [41].

There are three limitations in the current study. First of all, although numerous studies have demonstrated that the reactivity profile of the rat tail artery resembles the human peripheral arteries [42–44] and have been used as an *ex vivo* model of resistance artery which can be extrapolated to human peripheral vascular disease [45], quantitative measurement of cerebrovascular reactivity should be employed in the future study. In addition, while our current work showed that acute SLT administration did not induce endothelium-dependent vasodilatation in the vessels isolated from a healthy animal, previous work from our group has demonstrated that SLT protected the cultured human endothelial cell from oxidative injury [12], indicating that the endothelium modulatory effects of SLT can only be observed under diseased conditions. Hence, endothelial effects of SLT in disease models should be studied in the future. Finally, while the active components of SLT have been shown to be absorbed into systematic circulation [46], any effects mediated by the metabolites of SLT may be overlooked by the *ex vivo* model used in the current study. A detailed metabolomics study is required to identify and explore the possible contribution of metabolites of SLT on vasculature.

## 5. Conclusions

In conclusion, we demonstrated that acute administration of the SLT extract induced vasodilatation in rat isolated tail artery. SLT-induced vasodilatation appeared to be principally mediated *via* endothelium-independent mechanisms, including blockage of extracellular Ca^2+^ influx and inhibition of release of Ca^2+^ from the sarcoplasmic reticulum. Results from this work provide direct evidence to explain the beneficial effects of SLT observed in clinical trials.

## Data Availability

The data that support the findings of this study are available from the corresponding author (SWS) upon reasonable request.

## Abbreviations

SLT: Sailuotong
VaD: Vascular dementia
WKY: Wistar Kyoto
Ca^2+^: Calcium ion
K^+^: Potassium ion
KCl: Potassium chloride
CaCl_2_: Calcium chloride
SOD: Superoxide dismutase
CVD: Cardiovascular diseases
Ach: Acetylcholine
TEA: Tetraethylammonium
EGTA: Ethylene glycol-bis(*β*-aminoethyl ether)-*N*, *N*,*N*′,*N*′-tetraacetic acid
PE: Phenylephrine
L-NAME: *N*-nitroarginine methyl ester
K_ATP_: ATP-sensitive potassium channel
K_Ca_: Calcium-dependent potassium channel
SR: Sarcoplasmic reticulum
VSMC: Vascular smooth muscle cell
VOCC: Voltage-operate calcium channel
ROCC: Receptor-operate calcium channel.
